# Protective Effect and Mechanisms of New Gelatin on Chemotherapy-Induced Hematopoietic Injury Zebrafish Model

**DOI:** 10.1155/2019/8918943

**Published:** 2019-08-21

**Authors:** Liwen Han, Haotian Kong, Fasheng Liu, Xiaobin Li, Shanshan Zhang, Xuanming Zhang, Yong Wu, Hua Yang, Aiping Zhang, Kechun Liu

**Affiliations:** ^1^Biology Institute, Qilu University of Technology (Shandong Academy of Sciences), Engineering Research Center of Zebrafish Models for Human Diseases and Drug Screening of Shandong Province, Jinan, Shandong 250103, China; ^2^School of Pharmaceutical Science of Shanxi Medical University, Taiyuan, Shanxi 030001, China; ^3^Shandong Fupai Ejiao Co., Ltd., Jinan 250103, China

## Abstract

The aim of the study is to explore the protective effect of new gelatin (NG, Xin'ejiao in China) on hematopoietic injury caused by chemotherapy. Zebrafish, at 48 hours post fertilization (hpf), was treated with different chemotherapeutic drugs to establish the zebrafish hematopoietic damage model with reduced thrombocytes and erythrocytes. The protecting effects of NG on the thrombocytes and erythrocytes were observed, respectively, on zebrafish models. Then, the RT-PCR method was used to detect the change of mRNA level of the hematopoiesis-related cytokines *scl1*, *c-myb*, *pu.1*, *GATA1*, and *runx1* genes. The results showed that 50 *μ*g·mL^−1^ and 100 *μ*g·mL^−1^ NG rescued and increased the thrombocytes numbers induced by vinorelbine (NVB) and chloramphenicol (CHL) and the erythrocytes numbers induced by methotrexate (MTX), doxorubicin (ADM), and mechlorethamine hydrochloride (MH) in zebrafish models. Meanwhile, the mRNA expression of *scl1*, *c-myb*, and *GATA1* genes in the NG treatment group was raised compared with the MTX treatment group. Also, the mRNA expression of *pu.1* and *Runx1* in the NG treatment group was reduced compared with the MTX treatment group. In consequence, traditional Chinese medicine NG showed a certain degree protective effect on hematopoiesis injury induced by chemotherapy in this study, which may depend on the promotion of erythrocytes proliferation and the regulation of the hematopoietic genes level.

## 1. Introduction

As a traditional Chinese medicine (TCM), Colla corii asini (CCA, Ejiao) is a gelatin-like preparation derived from donkey hide. It has been widely used for sedation, anticoagulation, vasodilatation, and hematopoiesis, as well as the improvement in cellular immunity and radioprotection in Asia [1]. However, with the rapid increase in the use of donkey-derived gelatin, the shortage of donkey skin has become the main obstacle. Therefore, the development and research of gelatin derived from other animal skins is of great significance for the wide market application of Ejiao.

New gelatin (NG, Xin'ejiao in China) is a solid gelatin made from the skin of pig (*Sus scrofa domestica Brisson*). It is produced in only one company in China. According to TCM theory, the NG has the effect of nourishing yin deficiency, nourishing blood, and stopping bleeding. It can be used to treat weak anemia, menstrual disorders, vomiting blood, and blood stasis. So far, there is no adequate modern pharmacological research of NG, which restricts its clinical applications.

Chemotherapy is widely used for clinical treatment of tumors. Most antitumor drugs often cause injury of healthy tissues and cells while killing tumor cells. The hematopoietic system is one of common targets of chemotherapy [2]. Recently, the role of TCM in relieving chemotherapy side effects and improving the quality of life of cancer patients has attracted widespread attention of experts at home and from abroad [3]. It has been reported that cancer patients who took Ejiao after chemotherapy exhibited improvement in bone marrow transplantation and leukopenia [4]. However, little is known whether NG has similar effect of improving hematopoietic function of chemotherapy patients.

Zebrafish is a common small tropical fish with an adult body length of about 3–5 cm. Previous studies have shown that the genome of zebrafish is 87% homologous to humans. It has the advantages of small size, rapid propagation with a large amount of production, early embryo transparent in vitro, and a similar hematopoietic system as humans. It is another important vertebrate model after mice, which has been widely used in the field of life science research. Small molecules and drugs can easily penetrate embryos of zebrafish, and large-scale drug screening can be performed by simply soaking with zebrafish [5, 6]. Therefore, zebrafish has become a reliable animal model for studying the human hematopoietic system and related diseases. These models can be employed for examination of etiology of the diseases at the molecular and cellular levels and testing the effectiveness of screened drugs [7]. In this paper, the zebrafish model was used to study the pharmacological effects of NG in the treatment of hematopoietic injury caused by chemotherapy drugs, which may identify the scientific evidence for clinical application of NG.

## 2. Materials and Methods

### 2.1. Reagents

The following reagents were used: NG (Shandong Fujiao Group, Donga Town Ejiao Co., Ltd, lot: 15060022); vinorelbine (Beijing Century Aoke Biotechnology Co., Ltd., lot: 170410); chloramphenicol (Genvien, USA, lot: 6428010150); methotrexate (China Food and Drug Control Institute, lot: 100138–201606); mechlorethamine hydrochloride (Shanghai Aladdin Biochemical Technology Co. Ltd., lot: G1414051); doxorubicin (China Food and Drug Control Institute, lot: 130509–201302); Trizol (Coolaber, lot: SL2075); HiScript II Q RT SuperMix for qPCR (+gDNA wiper) (Vazyme, Nanjing, China, lot: R123-01); and AceQ qPCR SYBR Green Master Mix (Vazyme, Nanjing, China, lot: Q111-01/02/03).

### 2.2. Zebrafish Maintenance

Adult zebrafish were raised in the Key Laboratory of Drug Screening Technology of Shandong Academy of Sciences. The maintenance of zebrafish was done in accordance with Economic Cooperation and Development (OECD). The adult zebrafish AB strain and the Tg (CD41 : EGFP) transgenic zebrafish lines were used in this study. The light and dark alternate culture was adopted, and it was kept in the light for 14 h after 10 h, and the cycle temperature was controlled at 28 ± 0.5°C by the air-conditioner system to ensure normal spawning. Before the experiment, the female and male zebrafish were placed in the oviposition tank in proportion to 1 : 1 or 1 : 2 and were separated by the partition board. After drawing off the partition before the illumination the next day, the female and male zebrafish chased each other after contact and spawned. The embryos were collected within 1 h after the partition board was removed and were incubated in light incubator at 28°C.

### 2.3. Effect of NG on the Number of Thrombocytes Induced by Chemotherapeutic Drugs in Zebrafish

The Tg (CD41 : EGFP) zebrafish embryos developed to 48 hpf after fertilization were treated with 1 mg·mL^−1^ chain protease to remove the egg membrane. Then, the zebrafish embryos were randomly placed into 24-well plates at a density of 10 per well and were treated with vinorelbine (NVB) and chloramphenicol (CHL), respectively, as hematopoietic injury model. At the same time, each pore was added different doses of NG and nutrient solution to 2 mL; then, the final concentration of NVB and CHL was 100 and 150 *μ*g·mL^−1^ and the NG was 25, 50, and 100 *μ*g·mL^−1^. A 0.5% DMSO solvent group was used as the control group, while all the experimental groups were set up in three parallel groups and placed in a light incubator with the lid covering and incubated under the temperature and light control. After 24 hours, the images of each embryo was observed and collected by fluorescence microscope, and thrombocytes in the cloaca to tail region of zebrafish were counted by Image-Pro Plus software.

### 2.4. Effect of NG on the Number of Erythrocytes Induced by Chemotherapy Drugs in Zebrafish

The AB strain zebrafish at 24 hpf were treated with 1 mg·mL^−1^ chain protease to remove the egg membrane and were randomly placed into 24-well plates at a density of 30 per well. They were randomly divided into the solvent control group and the model group (methotrexate with 600 *μ*g·mL^−1^, doxorubicin with 150 *μ*g·mL^−1^, and mechlorethamine hydrochloride with 150 *μ*g·mL^−1^), and the model module (methotrexate 600 *μ*g·mL^−1^, doxorubicin with 150 *μ*g·mL^−1^, and mechlorethamine hydrochloride with 150 *μ*g·mL^−1^) different concentrations of NG group (25, 50, and 100 *μ*g·mL^−1^) were processed together. At 24 h after exposure, the erythrocytes of zebrafish were stained by o-Dianisidine in dark. After 15 min, the embryos were washed three times, and the specimens were photographed by a microscope. The number of erythrocytes of the yolk sac of zebrafish larvae was observed and quantified according to the staining intensity of erythrocytes in the yolk sac (SI) and analyzed by Image-Pro Plus 5.1 [8]. The staining intensity can be measured by integral optical density (IOD). In order to reduce the interference of melanin in yolk sac in zebrafish, it is necessary to add thiourea when the zebrafish develops to 12 hpf to inhibit melanin production.

### 2.5. Quantitative Real-Time Reverse Transcription PCR (qRT-PCR)

Total RNA was extracted from 40 zebrafish larvae using Trizol reagent (Invitrogen, USA), and cDNA was synthesized by reverse transcription using HiScript II Q RT SuperMix. cDNA was amplified by LightCycler® 96 fluorescence quantitative PCR. The amplification program of RT-PCR was as follows: denaturation at 95°C for 5 min, and then at 95°C for 10 s and 55°C for 30 s, with a total of 45 cycles. PCR instrument automatically collected the fluorescence signals after each cycle annealing. In the calculation of gene expression, *ß*-actin was used as an internal reference gene, and the fold-change of the genes tested was assessed using the 2-ΔΔCt method [9]. The primer sequence is shown in Table 1.

### 2.6. Data Analysis

The data were analyzed by the SPSS 18 software, and all data were expressed in $\bar{x}\pm s$. The Kolmogorov–Smirnov test is used to test the experimental data to investigate whether the data conform to the normal distribution, and when the data do not show the normal distribution, the data are converted to the normal distribution by logarithmic transformation. One-way ANOVA was used to analyze and compare data of different treatment groups. The LSD method was used to compare the two groups, and test standard *α* = 0.05 (bilateral).

## 3. Result

### 3.1. Effect of NG on Thrombocytes Formation in Zebrafish after Treatment with Chemotherapeutic Drugs

Tg (CD41 : EGFP) transgenic zebrafish was marked by green fluorescent protein (GFP) in CD41, which showed a green fluorescence under the fluorescence microscope. The CD41-GFP^+^ cells were corresponding to the number of thrombocytes in zebrafish.

**Figure 1:**
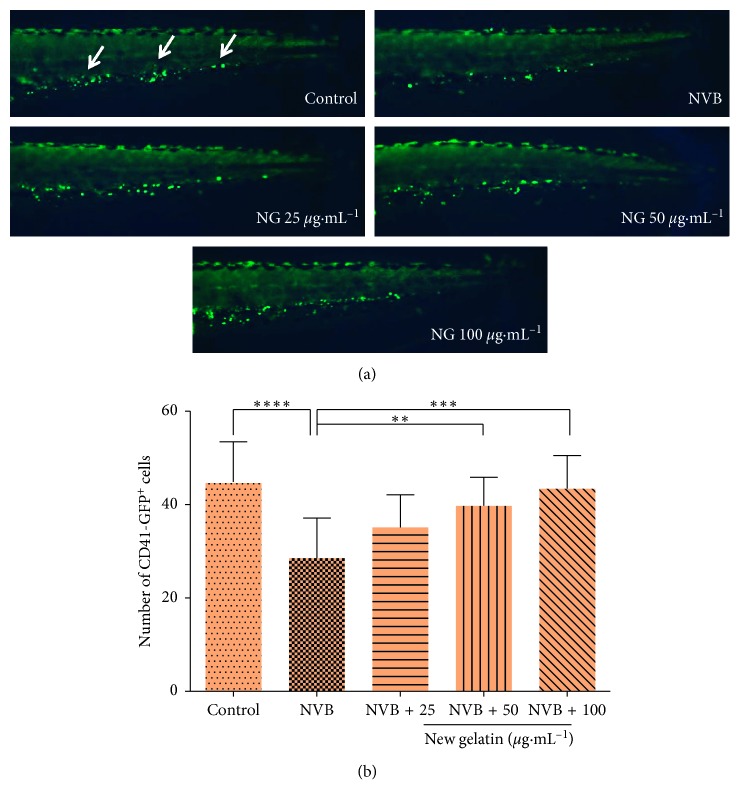
Effect of NG on the number of thrombocytes in zebrafish induced by vinorelbine (NVB). The number of CD41-GFP^+^ cells was counted in the caudal hematopoietic tissue area of zebrafish. (a) Phenotypes of larvae of Tg (CD41 : EGFP) lines. (b) Number of CD41-GFP^+^ cells at 72 hpf. The values are expressed as mean ± SEM (*n* = 10). ^*∗∗∗∗*^*P* < 0.001 versus Ctl, ^*∗∗*^*P* < 0.01, ^*∗∗∗*^*P* < 0.001 versus NVB.

The thrombocytes of zebrafish in vinorelbine module are shown in Figure 1, which showed that compared with the control group, the number and fluorescence intensity of the CD41-GFP^+^ cells in the caudal hematopoietic tissue of the zebrafish in the vinorelbine model group were significantly decreased, indicating that the vinorelbine affected the formation of thrombocytes in zebrafish and damaged the hematopoietic system of zebrafish in a certain range. With the addition of different doses of NG, the number of CD41-GFP^+^ cells increased gradually, and the number of CD41-GFP^+^ cells increased with the increase of the concentration of NG, showing a significant dose-dependent effect, indicating NG has a strong repair effect on the decrease of thrombocytes caused by vinorelbine.

**Figure 2:**
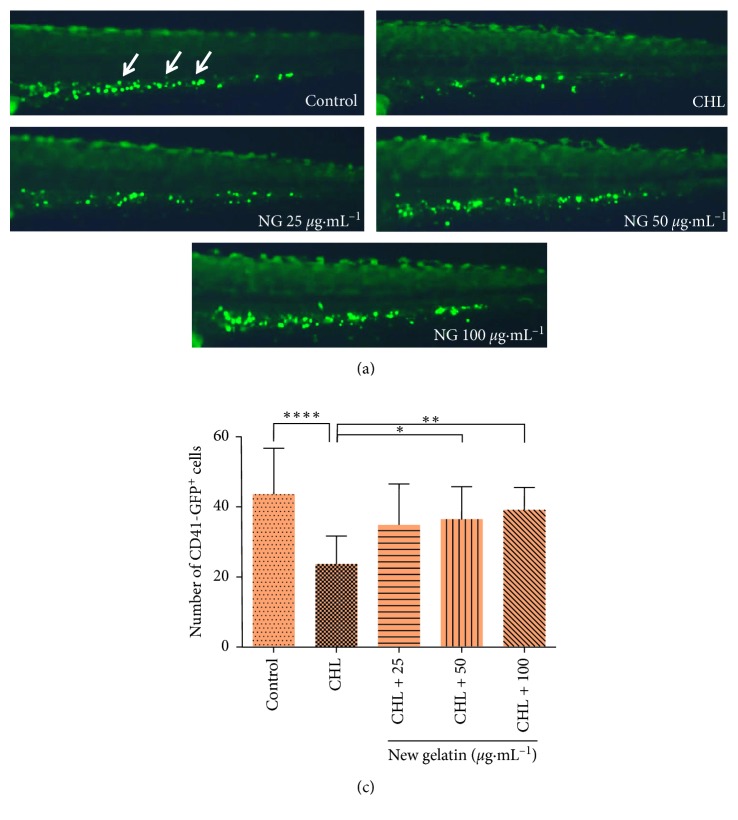
Effect of NG on the number of thrombocytes in zebrafish induced by chloramphenicol (CHL). The number of CD41-GFP^+^ cells was counted in the caudal hematopoietic tissue area of zebrafish. (a) Phenotypes of larvae of Tg (CD41 : EGFP) lines. (b) Number of CD41-GFP^+^ cells at 72 hpf. The values are expressed as mean ± SEM (*n* = 10). ^*∗∗∗∗*^*P* < 0.001 versus Ctl, ^*∗∗*^*P* < 0.01, ^*∗∗∗*^*P* < 0.001 versus CHL.

Similarly, after chloramphenicol treatment, the formation of CD41-GFP^+^ cells in zebrafish was also inhibited, and the number of CD41-GFP^+^ cells decreased significantly (Figure 2). After the treatment of different concentrations of NG, the number of CD41-GFP^+^ cells increased. Compared with the model group, the 25 *μ*g·mL^−1^ NG had statistical difference, and there was a significant dose-dependent effect in the concentration range of 25–100 *μ*g·mL^−1^.

### 3.2. Effect of NG on the Erythrocyte Production of Zebrafish after Treatment with Chemotherapeutic Drugs

**Figure 3:**
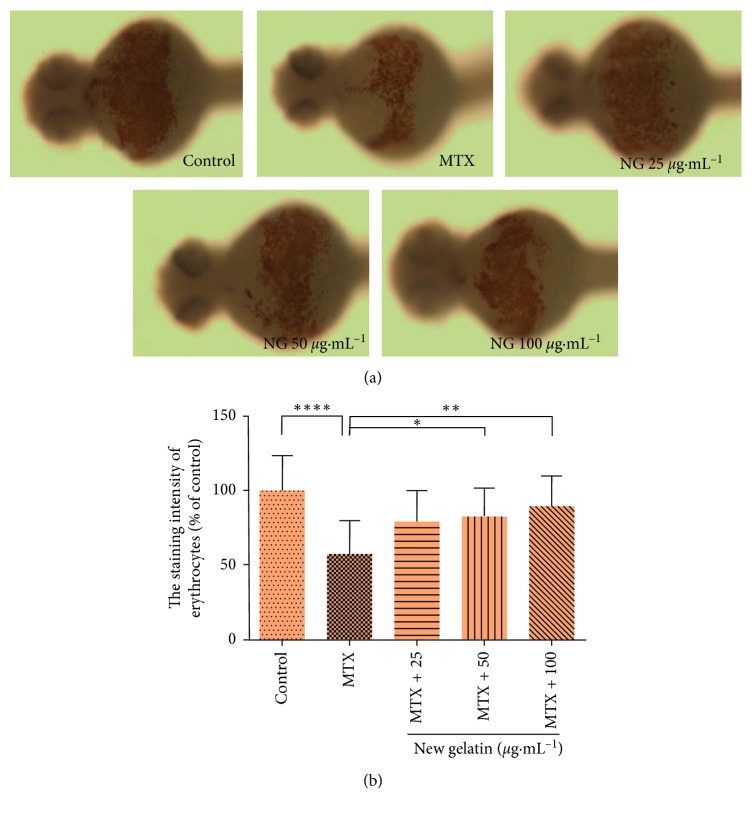
Effect of NG on the number of erythrocytes in zebrafish induced by methotrexate (MTX). (a) Phenotypes of larvae of wild-type AB lines. (b) The staining intensity of erythrocytes at 48 hpf. The values are expressed as mean ± SEM (*n* = 30). ^*∗∗∗∗*^*P* < 0.001 versus Ctl, ^*∗*^*P* < 0.05, ^*∗∗*^*P* < 0.01 versus MTX.

Methotrexate is an antifolic acid antitumor drug.  Figure 3 shows that the number of erythrocytes in juvenile zebrafish decreased significantly after treatment with 600 *μ*g·mL^−1^ methotrexate for 24 h and decreased to 46.2% compared with the control group, which has a significant statistical difference. After the treatment of NG, it was found that the content of erythrocytes in zebrafish increased significantly with the increase of the dose of NG. When the dose of NG was 50 *μ*g·mL^−1^, the erythrocytes content reached 75.6% compared with the methotrexate model, which increased by 54.6%, indicating that the NG has a good therapeutic effect on the reduction of the number of erythrocytes caused by methotrexate.

**Figure 4:**
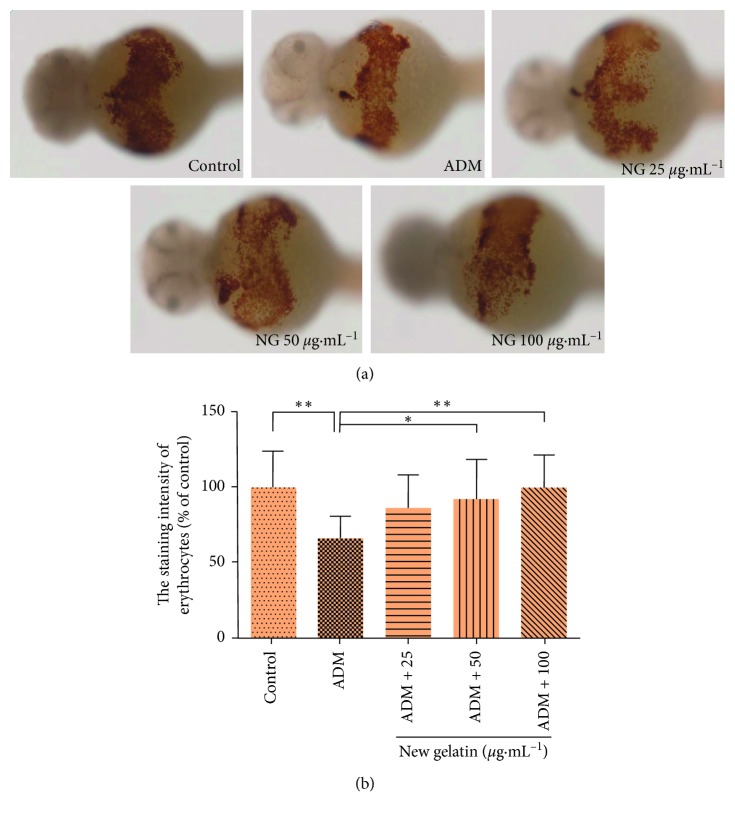
Effect of NG on the number of erythrocytes in zebrafish induced by doxorubicin (ADM). (a) Phenotypes of larvae of wild-type AB lines. (b) The staining intensity of erythrocytes at 48 hpf. The values are expressed as mean ± SEM (*n* = 30). ^*∗∗*^*P* < 0.01 versus Ctl, ^*∗*^*P* < 0.05, ^*∗∗*^*P* < 0.01 versus ADM.

The amount of erythrocytes in zebrafish after treatment with doxorubicin is shown in Figure 4. The doxorubicin of 150 *μ*g·mL^−1^ significantly inhibited the formation of erythrocytes in zebrafish, and the number of erythrocytes in zebrafish increased with the increase of the concentration of the NG.

**Figure 5:**
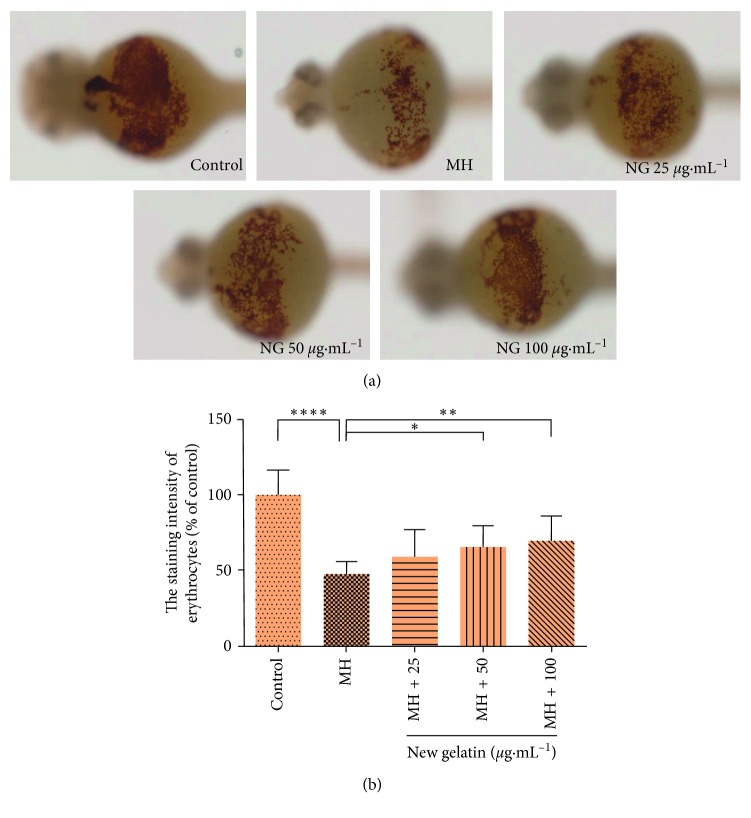
Effect of NG on the number of erythrocytes in zebrafish induced by mechlorethamine hydrochloride (MH). (a) Phenotypes of larvae of wild-type AB lines. (b) The staining intensity of erythrocytes at 48 hpf. The values are expressed as mean ± SEM (*n* = 30). ^*∗∗∗∗*^*P* < 0.0001 versus Ctl, ^*∗*^*P* < 0.05, ^*∗∗*^*P* < 0.01 versus MH.

The effect of mechlorethamine hydrochloride hydrochloride on erythrocytes of zebrafish is shown in Figure 5. The erythrocytes content of zebrafish decreased significantly after treatment with 150 *μ*g·mL^−1^ mechlorethamine hydrochloride, which indicated that mechlorethamine hydrochloride interfered the formation of erythrocytes in zebrafish. After the treatment of different concentrations of NG, the staining intensity of erythrocytes in the yolk sac was increased, indicating the increasing number of erythrocytes.

### 3.3. Effect of NG on the Expression of Hematopoietic-Related Factors in Zebrafish

**Figure 6:**
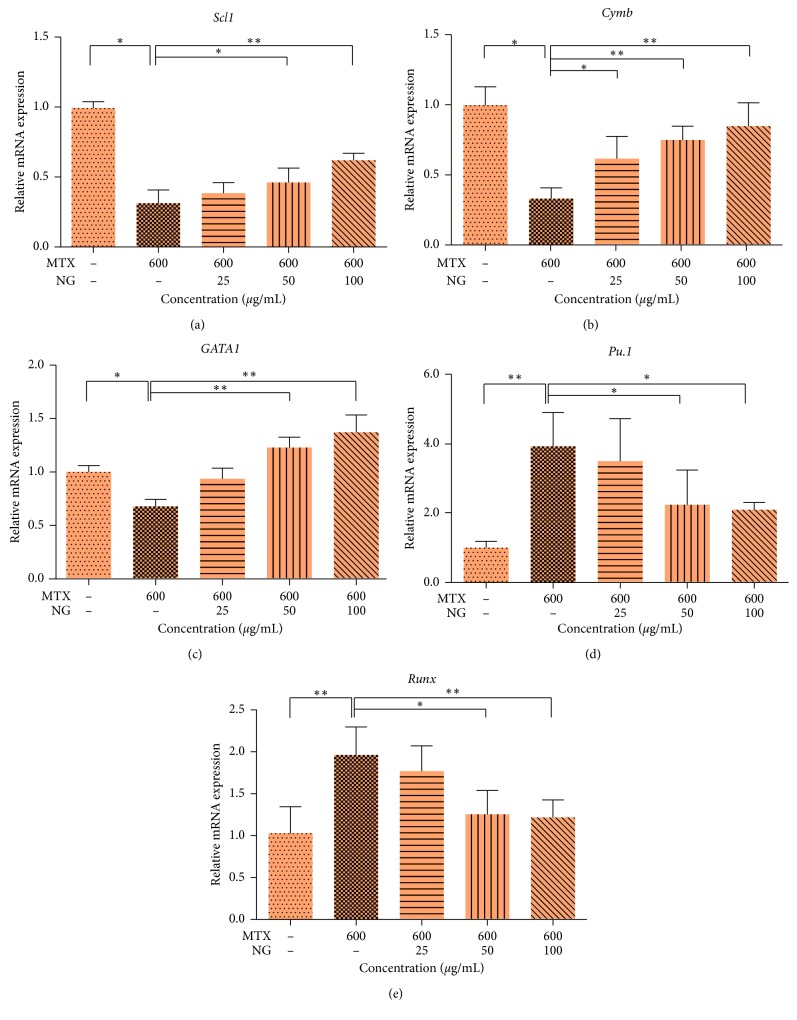
Expression of related genes in zebrafish hematopoietic injury model pretreated by methotrexate (MTX). The mRNA levels of *scl1* (a), *c-myb* (b), *GATA1* (c), *pu.1* (d), and *Runx1* (e). The values are expressed as mean ± SEM (*n* = 30). ^*∗*^*P* < 0.05, ^*∗∗*^*P* < 0.01 versus Ctl. ^*∗*^*P* < 0.05, ^*∗∗*^*P* < 0.01, ^*∗∗∗*^*P* < 0.001 versus MTX.

As shown in Figure 6, after 600 *μ*g·mL^−1^ methotrexate treatment, compared with the control group, the mRNA expression of hematopoietic stem cell generation related genes such as *scl1* and *GATA1* affecting hematopoietic stem cells differentiating between erythrocytes and transcription factor *c-myb* gene decreased, while the expression of *pu.1* gene that affected the differentiation of hematopoietic stem cells to the myeloid cells and the transcription factor *Runx1* gene increased significantly in the process of hematopoiesis. After the treatment of NG, the expression of *scl1*, *GATA1*, and *c-myb* increased in a dose-dependent manner, while the expression of *pu.1* and *Runx1* genes decreased.

## 4. Discussion

Bone marrow is the most important hematopoietic organ in the hematopoietic system. It is composed of hematopoietic cells at different mature stages such as erythrocytes, leukocytes, and platelets. Bone marrow hemopoietic tissue is so active in cell division that it is very sensitive to radiation and chemotherapeutic agents. Myelosuppression induced by radio therapy and chemotherapy is a big problem restricting the therapeutic effect of tumor. Thus, in order to enhance the therapeutic effect and improve the prognosis with no low side effects, seeking the drugs which can alleviate the myelosuppression caused by chemoradiotherapy is very crucial and challenging [10].

As a representative bulk Chinese medicine product, the price for Ejiao has been rising in recent years. It has become a very expensive Chinese medicine product. NG, the introduction of Ejiao alternatives, which is made from animal skin, has stirred up a strong public interest. It is of great significance to study the pharmacological effects of gelatin products from different skin sources by scientific methods for the correct clinical application of different types of gelatin. Based on the TCM theory, NG and Ejiao are expected to show similar efficacy and can be used interchangeably in most cases. There are many reports about Ejiao in the literature, but reports about NG are very few. From the point of view of hematopoietic injury induced by chemotherapy, the protective effect of NG on hematopoietic injury was explored in this paper. Studies have shown that there is no significant difference in free amino acids and total amino acids and trace elements between Ejiao and NG. This seems to simply confirm that NG and Ejiao have similar effects [11]. In a study of regulation of Ejiao and NG on hematopoietic injury induced by ^60^Co irradiation in mice, Xia found that the treatment effect of NG on hematopoietic injury was better than Ejiao [12]. The results of this study are consistent with the results of NG in reversing hematopoietic injury, which further confirm the blood-replenishing effect of NG. Meanwhile, some researchers used NG to treat U937 cells. It was found that the secretion of proinflammatory cytokines related to NF-κB increased, and the phagocytic function of macrophages differentiated from U937 cells was also enhanced [13]. Although NG had a good nourishing effect in the known studies, there are still great differences in it before a large amount of clinical application evidence is obtained. Based on this, we use the new animal zebrafish model for the first time to quickly verify the hematopoietic effect of the NG and provide experimental basis for the antitumor clinical application of NG.

As a high throughput drug screening model, zebrafish embryos and larvae fill the gap in the transition between cell model and rodent model. In order to find compounds that increase or decrease the number of hematopoietic stem cells (HSCs), North used automatic in situ hybridization to stain HSCs in the aortic-gonadal-mesonephric region of zebrafish embryos and found that PGE2 could increase the number of HSCs. The ability of PGE2 to enhance production of HSCs was later tied to its interaction with WNT signalling [14]. In addition, Jinchao et al. used the zebrafish model and found that platelet number was effectively increased by rhTyrRS (Y341A) via platelet count and reticulated platelets flow cytometry and demonstrated that radiation-induced thrombocytopenia could be prevented by rhTyrRS (Y341A) [15]. The above studies show that the zebrafish model has been increasingly recognized by researchers to explore the mechanism of drug protection.

The hemopoietic system of zebrafish is similar to humans and other advanced mammals. It has complete hematopoietic system, including erythroid, medullary, gonorrhea, and megakaryocyte. Its signal transduction pathway and related transcription factors are highly homologous with human beings. The hematopoietic genetic network is highly conservative in evolution. Primitive hematopoiesis of zebrafish mainly occurs in two extraembryonic regions, intermediate cell mass (ICM) and posterior mesoderm (ALPM) [16, 17]. The former is located between the chorda and trunk mesoderm, which functions in accordance with the islands of yolk sac in mammalian embryonic period, and the latter is the region of origin of early macrophages [18]. The hemopoietic system of humans and zebrafish is divided into primitive hematopoiesis and terminal hematopoiesis. With the beginning of blood circulation, the hematopoiesis in ICM decreases gradually, which indicates the end of primordial hematopoiesis. In the process of terminal hematopoiesis, endothelial cells are separated from peripheral cells through the aorta-gonad-mesonephric region (AGM) to enter the blood circulation, and then the hematopoietic stem cells are formed, which lays the foundation for the production of all kinds of erythrocytes in the hematopoiesis system. In addition, zebrafish embryos are transparent and hemoglobin is red, and the circulation of blood can be observed by the naked eye. A transgenic reporter zebrafish using the CD41 promoter to drive enhanced green fluorescent protein (EGFP) expression was previously generated. CD41-GFP^+^ cells expresses GFP in thrombocytes, thrombocyte precursors, and possibly early hematopoietic stem cells in zebrafish embryos [19]. Among these, CD41-EGFP^+^ cells' thrombocytes are the largest number ones. Although we are not sure whether CD41-GFP^+^ cells include hematopoietic stem cells, the results can show that thrombocytes are induced to increase or decrease by the drug. Therefore, we used the zebrafish model to study the protective effect of NG on hemopoietic inhibition.

Vinorelbine, chloramphenicol, methotrexate, doxorubicin, and cyclophosphamide are all first-line antitumor drugs in clinic. However, these chemotherapeutic drugs lead to myelosuppression, which limits the dose enhancement of chemotherapy and hinders the optimal treatment of cancer patients. On the basis of previous research, we found that experimental hematopoietic injury models are usually induced by chemotherapy or irradiation and are widely used in pharmacodynamic evaluation of hematopoiesis [6, 20–24]. Vinorelbine and chloramphenicol can cause hematopoietic disorders and reduce the number of platelets in mice [25, 26]. At the same time, chloramphenicol can produce oxidative stress to induce platelet apoptosis [27, 28]. Cyclophosphamide is an oral inactive prodrug that produces mechlorethamine hydrochloride through metabolism in vivo, which has a toxic effect on the cells of the human hematopoiesis system and easily causes thrombocytopenia, leukopenia, and anemia [29]. Therefore, mechlorethamine hydrochloride was directly used to treat zebrafish larvae to establish the hematopoietic injury model. It has been proved that these chemotherapeutic drugs can cause hematopoietic injury in zebrafish and mice.

In order to further explore the possible protective mechanism of NG on hemopoietic injury in zebrafish, we searched for genes that play an important regulatory role in zebrafish hematopoietic process to verify. *scl1*, *c-myb*, and *runx1* can regulate the differentiation of hematopoietic stem cells, while *pu.1* can regulate the development of myeloid cells and *GATA-1* can regulate the development of erythroid cells [30]. The formation and improvement of hematopoietic system is established by the gradual differentiation of stem cells into different hematopoietic cells under the regulation of hematopoietic transcription factors, and its functional complexity increases with the degree of differentiation [16, 31]. The results showed that methotrexate could affect the expression of *scl1*, *GATA-1*, and *c-myb* in hematopoietic progenitor cells, reduce the production of erythrocyte and megakaryocyte, and significantly increase the expression of *pu.1* and *Runx1* genes, indicating that zebrafish hematopoietic injury model was successfully established. Knockout of *scl1* gene in zebrafish will result in complete loss of primitive erythropoiesis and bone marrow cell production, as well as loss of expression of *c-myb* and *Runx1* in dorsal aorta, which indicates that it is very important in hematopoietic function [32–34]. Burns et al. [35] emphasized the hypothesis that *Runx1* is necessary in primary hematopoiesis, but also in final hematopoiesis. *Runx1* is considered to be one of the earliest markers of HSCs, and *c-myb* acts downstream. The analysis of zebrafish mutants shows that the expression of *c-myb* depends on *Runx1* [36]. The deletion of *c-myb* gene inhibits the development of erythrocyte in mice and then leads to anemia in mice [37]. Some studies have shown that for adult mice, *Runx1* deficiency shows mild myeloproliferative phenotype, and the number of neutrophils and bone marrow progenitor cell group in peripheral blood increases [38], and the composition of mature bone marrow and erythrocyte also increases, which indicates that different hematopoietic factors have different effects at different stages of hematopoietic progenitor cells or hematopoietic lineage development. *GATA1* deficiency will lead to abnormal cell apoptosis in the early proliferation and differentiation of erythrocytes, leading to severe anemia and severe thrombocytopenia [35, 36, 39], and *GATA1* plays an indispensable role in determining the fate of bone marrow-erythrocytic lineage during embryogenesis [40]. Unlike *GATA1*, *pu.1* can regulate the development of hematopoietic stem cells into myeloid cells, forming lymphoid bone marrow progenitor cells, and further differentiate into immune cells such as granulocytes, macrophages, lymphocytes, and so on under the action of downstream regulatory factors. There were antagonistic effects between *GATA1* and *pu.1*, which maintained the dynamic balance of hematopoiesis differentiation [40–42]. Therefore, we selected the above five key genes to study the potential mechanism of the reversal of hematopoietic injury by NG. After treatment with NG, the expression of *scl1*, *GATA1*, and *c-myb* increased with the increase of drug concentration, while the expression of *pu.1* and *runx1* genes showed a decreasing trend, indicating that NG could improve the blood microenvironment, promote the proliferation of hematopoietic stem cells, and inhibit the apoptosis of hematopoietic mother cells, thereby promoting the formation of hematopoietic cells and having the function of hematopoietic protection [43].

## 5. Conclusion

The zebrafish model provides a huge balance between scale and applicability. Based on this model, NG has the protective effect on hematopoietic injury induced by chemotherapy, which is shown by its reversal effect on thrombocytopenia and erythrocyte reduction induced by chemotherapeutic drugs. Its mechanism may be related to the promotion of the expression of key hematopoietic factors.

## Figures and Tables

**Table 1:** Primers used in real-time PCR.

Gene	Primer orientation	Sequence of primer (5′-3′)
*β-Actin*	Forward	GAACCGCTGCCTCTTCTTCCTCC
	Reverse	CCCTGTTAGACAACTACCTCCCTTT
*runx1*	Forward	ACATATCAGAGAGCCATAAAG
	Reverse	CAGATATGGATACGACTGC
*scl*	Forward	TCCCAGAGACCCGCTGAGCG
	Reverse	CAGGAGGGTGTGTTGGGATG
*pu.1*	Forward	AGAGAGGGTAACCTGGACTG
	Reverse	AAGTCCACTGGATGAATGTG
*c-myb*	Forward	TTTCTACCGAATCGAACAGATG
	Reverse	CAATCACCCGTTGGTCTTCT
*GATA1*	Forward	AAGATGGGACAGGCCACTAC
	Reverse	TGCTGACAATCAGCCTCTTTT

## Data Availability

The data used to support the findings of this study are available from the corresponding author upon request.
